# Cardiorenal syndrome in thalassemia patients

**DOI:** 10.1186/s12882-020-01990-8

**Published:** 2020-08-03

**Authors:** Sorasak Makmettakul, Adisak Tantiworawit, Arintaya Phrommintikul, Pokpong Piriyakhuntorn, Thanawat Rattanathammethee, Sasinee Hantrakool, Chatree Chai-Adisaksopha, Ekarat Rattarittamrong, Lalita Norasetthada, Kanda Fanhchaksai, Pimlak Charoenkwan, Suree Lekawanvijit

**Affiliations:** 1 Division of Hematology, Department of Internal Medicine, Faculty of Medicine, Chiang Mai University, 110 Intravaroros Road, A. Muang, Chiang Mai, 50200 Thailand; 2 Lerdsin Hospital, Bangkok, Thailand; 3 Division of Cardiology, Department of Internal Medicine, Faculty of Medicine, Chiang Mai University, Chiang Mai, Thailand; 4 Division of Hematology and Oncology, Department of Pediatrics, Faculty of Medicine, Chiang Mai University, Chiang Mai, Thailand; 5 Department of Pathology, Faculty of Medicine, Chiang Mai University, Chiang Mai, Thailand

**Keywords:** Cardiorenal syndrome, Thalassemia, Heart failure, Kidney failure

## Abstract

**Background:**

Cardiorenal syndrome (CRS), a serious condition with high morbidity and mortality, is characterized by the coexistence of cardiac abnormality and renal dysfunction. There is limited information about CRS in association thalassemia. This study aimed to investigate the prevalence of CRS in thalassemia patients and also associated risk factors.

**Methods:**

Thalassemia patients who attended the out-patient clinic of a tertiary care university hospital from October 2016 to September 2017 were enrolled onto this cross-sectional study. Clinical and laboratory findings from 2 consecutive visits, 3 months apart, were assessed. The criteria for diagnosis of CRS was based on a system proposed by Ronco and McCullough. Cardiac abnormalities are assessed by clinical presentation, establishment of acute or chronic heart failure using definitions from 2016 ESC guidelines or from structural abnormalities shown in an echocardiogram. Renal dysfunction was defined as chronic kidney disease according to the 2012 KDIGO guidelines.

**Results:**

Out of 90 thalassemia patients, 25 (27.8%) had CRS. The multivariable analysis showed a significant association between CRS and extramedullary hematopoiesis (EMH) (odds ratio (OR) 20.55, *p* = 0.016); thalassemia type [β^0^/β^E^ vs β^0^/β^0^ thalassemia (OR 0.005, *p* = 0.002)]; pulmonary hypertension (OR 178.1, *p* = 0.001); elevated serum NT-proBNP (OR 1.028, *p* = 0.022), and elevated 24-h urine magnesium (OR 1.913, *p* = 0.016). There was no association found between CRS and frequency of blood transfusion, serum ferritin, liver iron concentration, cardiac T2*, type of iron chelating agents, or urine neutrophil gelatinase-associated lipocalin level.

**Conclusions:**

CRS is relatively common in thalassemia patients. Its occurrence is associated with laboratory parameters which are easily measured in clinical practice.

## Background

Thalassemia, characterized by a mutation of a globin gene, is a common cause of congenital chronic hemolytic anemia in many parts of Southeast Asia including Thailand [1]. The severity of anemia varies by mutation type and the need for regular transfusion. The latter categorizes patients into two groups, transfusion dependent thalassemia (TDT) and non-transfusion dependent thalassemia (NTDT) [2–4]. The major cause of death in thalassemia is heart failure secondary to chronic iron overload, a condition known as iron overload cardiomyopathy. In the past, thalassemia patients with iron overload cardiomyopathy usually died within the 1^st^ or 2^nd^ decade of life [5–8]. However, an improvement of medical care and iron chelation therapy has led to a decrease in cardiac death and improved life expectancy in these patients [7–9]. Nevertheless, such patients are still at high risk of complications associated with chronic hemolytic anemia, especially those associated with heart and renal abnormalities.

Heart abnormalities are still mainly caused by chronic iron deposition in myocytes which results in an increase in oxidative stress, inducing myocyte injury, increased myocardial fibrosis and decreased cardiac contraction [10]. Renal abnormalities in cases of thalassemia manifest themselves through tubular dysfunction, glomerular dysfunction, hyperfiltration and renal stones caused by chronic hemolytic anemia and chelation therapy [11–17]. A coexistence of cardiac and renal abnormalities is known as ‘Cardiorenal syndrome (CRS)’. CRS has been defined by Ronco et al. [18] as abnormalities of heart and kidney which can be categorized into five types. Types 1 to 4 CRS, known as primary CRS, are caused by either a diseased heart or kidney whilst type 5 CRS, or secondary CRS, is defined as systemic conditions leading to simultaneous injury and/or dysfunction of both heart and kidney. Thalassemia is classified as a systemic disease so the simultaneous occurrence of cardiac and renal dysfunction is categorized as secondary, type 5 CRS. However, if the onset of cardiac and renal abnormalities differs, it could be categorized as any type of primary CRS (type 1–4). There is currently little information concerning CRS in thalassemia. Therefore, this study aimed to determine the prevalence of secondary CRS in thalassemia patients and its associated risk factors.

## Methods

### Patient population

This was a cross-sectional cohort study. All thalassemia patients, including a TDT and NTDT population, from the hematology out-patient clinic at Chiang Mai University Hospital were enrolled from October 2016 to September 2017. Inclusion criteria were to have an underlying condition of thalassemia and be aged > 18 years. Exclusion criteria were congenital renal or heart disease and patients who had missing or inadequate 24-h urine samples. Baseline characteristic data were collected. These included age, sex, thalassemia type, transfusion status, blood pressure, past medical history (in particular a history of diabetes, hypertension, dyslipidemia, thyroid and adrenal function), current medication and chelation therapy, iron status, previous cardiac T2* magnetic resonance imaging (MRI) and MRI for liver iron concentration (LIC), clinical history of heart failure, echocardiogram within 2 years (parameters including left ventricular ejection fraction, right ventricular systolic pressure, mean pulmonary arterial pressure, tricuspid velocity, diastolic dysfunction, E/A and E/E’ ratio of mitral valve) and any other complications. Clinical and laboratory assessments were performed on 2 consecutive visits which were at least 3 months apart. Laboratory tests included serum NT-proBNP, spot urine protein, spot urine albumin, spot urine electrolytes including calcium, 24-h urine protein, and 24-h urine electrolytes. This study was approved by the ethical review board of the Faculty of Medicine, Chiang Mai University (STUDY CODE: MED-2559-043461 Research ID: 4346, Approval number 046/2017). All the patients provided written informed consent.

### Definition and measurements

At the first visit, urine samples were collected from each patient for spot urine protein, albumin, creatinine, electrolytes and urinalysis. Blood samples were collected to test complete blood count (CBC), blood urea nitrogen (BUN), serum creatinine (SCr), electrolytes, ferritin, liver function test, serum NT-proBNP, lactate dehydrogenase (LDH), thyroid function test (TFT) and serum morning cortisol. A chest X-ray and electrocardiogram were also performed in all patients.

At the second visit, which took place at least 3 months after the first, the same lab tests were repeated and in addition 24-h urine samples were collected to measure 24-h urine protein, creatinine, and electrolytes. The sufficiency of 24-h urine collection was checked using urinary creatinine (Cr), sex and body weight and the following equations:

$$ \mathrm{Men}=\left(\mathrm{urinary}\ \mathrm{creatinine},\mathrm{mg}/\mathrm{d}\right)/\left(24\ \mathrm{x}\ \mathrm{body}\ \mathrm{weight}\right) $$

$$ \mathrm{Women}=\left(\mathrm{urinary}\ \mathrm{creatinine},\mathrm{mg}/\mathrm{d}\right)/\left(21\ \mathrm{x}\ \mathrm{body}\ \mathrm{weight}\right) $$

If the ratio of Cr to body weight (BW) was < 10.8 or > 25.2 and a total urine volume < 1000 mL/d with a urinary creatinine level < 5 mmol/d, the urine collection was deemed incomplete [19, 20].

We chose a follow up of at least 3 months to allow certainty as regards the definition and diagnostic criteria of chronic kidney disease. Estimated glomerular filtration rate (eGFR) was calculated using the CKD-EPI formula.

Kidney tubular injury was assessed by measuring urine NGAL. Spot urine samples (3 ml) from both visits of every patient were also stored at -80 °C to enable urine neutrophil gelatinase-associated lipocalin (NGAL) assessment. When all samples had been collected the measurement of urine NGAL levels were performed using a chemiluminescent microparticle immunoassay (ARCHITECT Urine NGAL assay, Longford, Ireland). Comparison of the automated ARCHITECT assay and a manual ELISA assay can be used to indicate a good correlation between the methods (Spearman’s rank correlation coefficient = 0.99) with a least squares linear regression line ARCHITECT = 0.93 (ELISA) + 4.2 (95% confidence interval for slope and intercept, 0.91 to 0.95 and − 0.8 to 9.2, respectively). The lower limit for detection NGAL of this assay is 1 ng/mL [21]. Chronic excretion of urine NGAL was defined as urine NGAL levels > 5 ng/ml at both visits.

NTDT is defined as thalassemia disease that does not require regular transfusion for survival. However, the definition of regular transfusion varies between studies. The criteria for NTDT used in this study were no more than three transfusions or 7 units of red cells in a year. TDT was defined as thalassemia that requires more than 3 transfusions a year and a transfusion free period of not more than 8 weeks and/or the number of red cell transfusions in the past year was greater than 7 units [4].

Extramedullary hematopoiesis (EMH) was diagnosed from chest X-ray and/or CT-scan and/or MRI.

In this study, CRS [18] was defined as the following:

1. Heart failure and/or cardiac abnormalities. The diagnostic criteria used to assess heart failure in this study were based on the 2016 European Society of Cardiology (ESC) Guidelines for the diagnosis and treatment of acute and chronic heart failure [22]. These criteria classify patients into heart failure with a preserved ejection fraction (HFpEF), mid-range ejection fraction (HFmrEF) and reduced ejection fraction (HFrEF) as shown in supplementary table S1. Typical symptoms of heart failure include breathlessness, orthopnea, paroxysmal nocturnal dyspnea, reduced exercise tolerance, fatigue, tiredness, increased time to recover after exercise, and ankle swelling. Typical signs of heart failure include elevated jugular venous pressure, hepatojugular reflux, third heart sound (gallop rhythm), and laterally displaced apical impulse.
  Cardiac abnormalities included structural remodeling identified by echocardiogram (such as left ventricular hypertrophy, cardiomegaly and iron overload cardiomyopathy or hemochromatosis) and cardiac dysfunction. Diagnostic criteria for left ventricular hypertrophy were based on QRS voltage criteria i.e. R wave in V5/V6 plus S wave in V1/V2 exceeds 35 mm in height (SV_1–2_ + RV_5–6_ > 35 mm). Diagnostic criteria for right ventricular hypertrophy were right axis deviation with tall R-waves in RV leads and deep S-waves in LV leads. Left atrial abnormality was defined as P-wave > 120 millisecond and wide notched P-wave > 40 milliseconds. Right atrial abnormality was defined as upright P-wave in lead II > 2.5 mm [23]. The criterion for diagnosis of cardiomegaly from a standard chest X-ray was a cardiothoracic ratio > 0.5 [24]. In accordance with Thalassemia International Federation guidelines, we defined hemochromatosis using the cardiac T2* technique and magnetic resonance imaging of the liver to determine liver iron concentration (LIC). Hemochromatosis was diagnosed when cardiac T2* was less than 20 milliseconds or the LIC more than 7 mgFe/g dry weight [3, 4].
2. Chronic kidney disease. The KDIGO 2012 Clinical Practice Guidelines for the Evaluation and Management of Chronic Kidney Disease [25] state that the condition is diagnosed if abnormalities of kidney structure or function are present for > 3 months. These include:

- GFR < 60 ml/min/1.73 m^2^ or
- Presence of 1 or more markers of kidney damage: albuminuria (Albumin excretion rate (AER) ≥30 mg/24 h; Albumin to creatinine ratio (ACR) ≥30 mg/g [≥3 mg/mmol]); urine sediment abnormalities; electrolyte and other abnormalities due to tubular disorders; abnormalities detected by histology; structural abnormalities, kidney stones detected by imaging; history of kidney transplantation.

### Statistical analysis

Statistical analysis was carried out using SPSS version 23.0. Sample size was calculated by estimation for a single proportion model. Previously published data on prevalence of renal and cardiac abnormalities was used to calculate sample size [26–28]. Estimated sample size was 90 for an alpha of 0.05 and power 0.8. Pearson’s Chi-square or Fisher’s exact test was performed to calculate the association between CRS and other sets of categorized data and to calculate odds ratios. Normality was checked using Kolmogorov–Smirnov and Shapiro-Wilk methods. The statistical significance of differences in continuous data sets was calculated using an independent sample t-test. Mann-Whitney U-test was used for non-normally distributed variables. Multivariable analysis was performed using a binary logistic-regression model which included all variables from the univariate logistic regression with significant differences. A *p* value of < 0.05 was considered significant.

## Results

Ninety thalassemia patients were enrolled onto this study, 75 (83.3%) classed as transfusion dependent thalassemia and 15 (16.7%) as non-transfusion dependent thalassemia. Twenty-five patients (27.8%) had coexisting cardiac and renal abnormalities or secondary CRS. Cardiac abnormalities were detected in 35 patients (38.9%), all had structural cardiac abnormalities and 8 had clinical heart failure. Renal abnormalities were detected in 52 patients (57.8%), 51 of these had chronic proteinuria alone and 1 had chronic proteinuria with eGFR below 60 mL/min (supplementary table S2). Eighty-eight patients (98.7%) had a complete 24-h urine collection.

The univariable analysis indicated that CRS was more frequently observed in β^0^/β^0^ thalassemia when compared with other types of thalassemia (*p* = 0.04) (Table 1). The detail of thalassemia type is provided in Table 2. Thalassemia with secondary CRS also showed a significant association with pulmonary hypertension (PHT) (*p* < 0.001), hemochromatosis (*p* = 0.047) and the presence of extramedullary hematopoiesis (EMH) (*p* = 0.02). In addition, patients with CRS had a significantly higher level of serum NT-proBNP, 24-h urine protein and 24-h urine magnesium than those without CRS.

**Table 1:** Baseline characteristics

	Total (*n* = 90)	CRS (*n* = 25)	No CRS (*n* = 65)	OR	*p*-value
Median age (yrs) (range)	29 (16–58)	30 (21–57)	28 (16–58)		0.256
Sex (%)				0.64	0.425
Male	31 (34.4)	7 (28.0)	24 (36.9)		
Female	59 (65.6)	18 (72.0)	41 (63.1)		
Mean arterial pressure (mmHg) (±SD)	76.3 (±8.8)	74.2 (±6.6)	77.1 (±9.5)		0.262
Diabetes mellitus (%)	5 (5.6)	2 (8.0)	3 (4.6)	1.80	0.615
Hypertension (%)	0	0	0		
Dyslipidemia (%)	0	0	0		
Hypothyroidism (%)	26 (28.9)	7 (28.0)	19 (29.2)	0.94	0.908
Adrenal insufficiency (%)	7 (7.8)	4 (16.0)	3 (4.6)	3.94	0.09
Thalassemia type (%)					
β^0^/β^E^ thalassemia	57 (63.3)	11 (44.0)	46 (70.8)	0.325	0.018
β^0^/β^0^ thalassemia	26 (28.9)	12 (48.0)	14 (21.5)	3.36	0.013
HemoglobinH disease	7 (7.8)	2 (8.0)	5 (7.7)	1.043	0.961
Pre-transfusion Hemoglobin (g/dL) mean (±SD)	7.25 (±1.04)	7.18 (±1.14)	7.28 (±1.01)		0.70
Splenectomy (%)				2.71	0.055
Yes	54 (60.0)	19 (76.0)	35 (53.8)		
No	36 (40.0)	6 (24.0)	30 (46.2)		
Transfusion-dependent (%)				2.88	0.219
Yes	75 (83.3)	23 (92.0)	52 (80.0)		
No	15 (16.7)	2 (8.0)	13 (20.0)		
Presence of EMH (%)				4.59	0.02
Yes	31 (34.4)	15 (60.0)	16 (24.6)		
No	59 (65.6)	10 (40.0)	49 (75.4)		
Ferritin (ng/mL) mean (±SD)	1783.2 (±1313.7)	1577.6 (±1133.2)	1877.0 (±1376.7)		0.30
Hemochromatosis (%)				2.60	0.047
Yes	42 (46.7)	16 (64.0)	26 (40.0)		
No	48 (53.3)	9 (36.0)	39 (60.0)		
Mean liver iron concentration (mg/g) (±SD)	13.78 (±5.89)	13.26 (±6.24)	14.02 (±5.80)		0.70
Mean cardiac T2 star (ms) (±SD)	37.20 (±12.23)	36.76 (±16.37)	37.42 (±9.98)		0.96
Type of iron chelation (%)					
Deferoxamine (DFO)	41 (45.6)	12 (48.0)	29 (44.6)	1.15	0.773
Deferiprone (DFP)	65 (72.2)	18 (72.0)	47 (61.5)	0.99	0.977
Deferasirox (DFX)	13 (14.4)	2 (8.0)	11 (16.9)	0.43	0.281
Combined DFO + DFP	25 (27.8)	7 (28.0)	18 (27.7)	1.01	0.977
Combined DFP + DFX	4 (4.4)	0 (0)	4 (6.2)	–	0.573
Presence of pulmonary hypertension (%)				25.50	< 0.001
Yes	17 (18.9)	14 (56.0)	3 (4.6)		
No	73 (81.1)	11 (44.0)	62 (95.4)		
Electrocardiogram (%)					0.138
Normal sinus rhythm	63 (70.0)	15 (60.0)	48 (73.9)		
Abnormal rhythm	7 (7.8)	1 (4.0)	6 (9.2)		
ST segment change	20 (22.2)	9 (36.0)	11 (16.9)		
Echocardiogram mean (±SD)					
Left ventricular ejection fraction [LVEF], (mmHg)		64.8 (±8.17)	65.0 (±4.67)		0.949
Right ventricular systolic pressure [RVSP], (mmHg)		67.1 (±35.68)	49 (±2.82)		0.509
Mean pulmonary arterial pressure [mPAP], (mmHg)		50.1 (±42.2)	23.0 (±5.0)		0.324
Tricuspid velocity [TV], (m/s)		329.7 (±95.7)	256.7 (±48.6)		0.090
E/A ratio of mitral valve		1.21 (±0.31)	1.55 (±0.54)		0.139
E/E’ ratio of mitral valve		11.9 (±2.2)	11.7 (±3.0)		0.899
Serum NT-proBNP (ng/ml) median (IQR)	137.01 (78.3–195.8)	141.6 (65.3–217.9)	99.47 (37.5–161.4)		0.028
Mean serum Creatinine (mg/dl) (±SD)	0.57 (±0.17)	0.55 (±0.2)	0.58 (±0.15)		0.37
Different in serum creatinine at 0 and 3 months (mg/dl) mean (±SD)	0.0689 (±0.055)	0.0688 (±0.057)	0.069 (±0.055)		1.00
24 h urine median (IQR)					
Protein (mg/24 h.)	193.0 (103.0-183.0)	273.0 (186.2–359.8)	175.0 (102.8–247.2)		< 0.001
Potassium (mmol/24 h.)	26.9 (17.8–36.0)	30.3 (19.0–41.6)	25.6 (17.4–33.9)		0.214
Phosphorus (mg/24 h.)	471.7 (309.4–634.1)	449.9 (213.3–686.5)	486.4 (332.5–640.5)		0.93
Magnesium (mEq/24 h.)	4.1 (2.8–5.4)	5.48 (3.9–7.0)	3.85 (2.7–5.0)		0.008
Calcium (mg/24 h.)	58.9 (13.1–104.7)	50.8 (1.8–99.8)	72.85 (26.6–119.1)		0.247
Mean urinary NGAL (ng/ml) median (IQR)	9.55 (3.8–15.3)	11.67 (3.5–19.9)	9.0 (4.4–13.6)		0.143
Chronic Urinary NGAL > 5 ng/ml (%)				2.82	0.038
Yes	49 (54.4)	18 (72.0)	31 (47.7)		
No	41 (45.6)	7 (28.0)	34 (52.3)		

*CRS* Cardiorenal syndrome, *OR* odd ratio, *EMH* extramedullary hematopoiesis, *yrs* years, *ms* millisecond

**Table 2:** Clinical features classified by type of thalassemia

	TDT (*n* = 75)	NTDT (*n* = 15)	*p*-value
Thalassemia type (%)			
β^0^/β^E^ thalassemia	48 (53.3)	9 (10.0)	
β^0^/β^0^ thalassemia	25 (27.8)	1 (1.1)	< 0.001
Hemoglobin H disease	2 (2.2)	5 (5.6)	
Sex (%)			
Male	26 (28.9)	5 (5.6)	0.921
Female	49 (54.4)	10 (11.1)	
Splenectomy (%)			
Yes	50 (55.6)	4 (4.4)	0.004
No	25 (27.8)	11 (12.2)	
Hemochromatosis (%)			
Yes	39 (43.8)	3 (3.4)	0.021
No	35 (39.3)	12 (13.5)	
Cardiorenal syndrome (%)			0.171
Yes	23 (25.6)	2 (2.2)	
No	52 (57.8)	13 (14.4)	
Mean ferritin (ng/ml) (±SD)	1785 (±1302)	1771 (±1417)	0.701
Pre-transfusion Hemoglobin (g/dL) mean(±SD)	7.15 (±0.96)	7.76 (±1.34)	0.039

*TDT* transfusion dependent thalassemia, *NTDT* non-transfusion dependent thalassemia

Patients with chronic excretion of urine NGAL at a level higher than 5 ng/ml showed a significant association with the occurrence of CRS in the univariable analysis (OR 2.82, *p* = 0.038) (Table 3), but not in the multivariable analysis (Table 4). Chronic excretion of urine NGAL > 5 ng/ml also showed a significant association with combined deferoxamine and deferiprone treatment (OR 3.69, *p* = 0.011), female gender (OR 5.15, *p* < 0.001), hemochromatosis (OR 3.01, *p* = 0.012), elevated serum LDH (OR 2.81, *p* = 0.018) and chronic proteinuria (OR 2.63, *p* = 0.025). The 24-h urine protein level was significantly higher in patients with chronic excretion of urine NGAL > 5 ng/ml than those without (*p* = 0.035).

**Table 3:** Chronic excretion of urinary NGAL

	Chronic excretion of urinary NGAL		OR	***p***-value
	Yes = 49 n (%)	No = 41 n (%)		
Sex			5.15	< 0.001
Male	9 (10.0)	22 (24.4)		
Female	40 (44.4)	19 (21.1)		
Thalassemia type				0.207
β^0^/β^E^ thalassemia	30 (33.3)	27 (30.0)		
β^0^/β^0^ thalassemia	15 (16.7)	11 (12.2)		
Hemoglobin H disease	4 (4.4)	3 (7.3)		
Splenectomy	29 (32.2)	25 (27.8)	0.92	0.055
Type of iron chelation				
Deferoxamine (DFO)	25 (27.8)	16 (17.8)	1.62	0.255
Deferiprone (DFP)	39 (33.3)	26 (28.8)	2.25	0.088
Deferasirox (DFX)	6 (6.7)	7 (7.8)	0.67	0.516
Combined DFO + DFP	19 (21.1)	6 (6.7)	3.69	0.011
Combined DFP + DFX	2 (2.1)	2 (2.1)	0.83	0.855
Presence of EMH	21 (23.3)	10 (11.1)	2.32	0.066
Presence of pulmonary hypertension	12 (13.3)	5 (5.6)	2.20	0.168
Presence of LVH	11 (12.2)	10 (11.1)	0.86	0.778
Presence of heart failure	7 (7.8)	1 (1.1)	6.67	0.067
Hemochromatosis	29 (32.2)	13 (14.4)	3.01	0.012
Elevated serum LDH	33 (36.7)	18 (20.0)	2.81	0.018
Elevated serum NT-proBNP	21 (23.3)	18 (20.0)	0.96	0.921
24 h urine, Median (IQR)				
Protein (mg/24 h.)	221.0 (143.4–298.6)	157.0 (80.3–233.8)		0.035
Sodium (mmol/24 h.)	126.0 (76.6–175.4)	127.0 (80.4–173.6)		0.471
Magnesium (mEq/24 h.)	4.9 (3.1–6.7)	3.8 (2.5–5.0)		0.094
Chronic Proteinuria	33 (36.7)	18 (20.0)	2.63	0.025
Cardiorenal syndrome	18 (20.0)	7 (7.8)	2.82	0.038
Mean liver iron concentration (mg/g) (±SD)	13.98 (±5.65)	13.4 (±6.34)		0.793
Mean cardiac T2 star (ms) (±SD)	38.0 (±14.1)	36.0 (±9.19)		0.591
Echocardiogram mean (±SD)				
Left ventricular ejection fraction [LVEF], (mmHg)	66.2 (±6.4)	62.1 (±8.4)		0.198
Right ventricular systolic pressure [RVSP], (mmHg)	56.0 (±17.7)	99.0 (±72.2)		0.093
Mean pulmonary arterial pressure [mPAP], (mmHg)	53.0 (±39.6)	18.2 (±0.4)		0.192
Tricuspid velocity [TV], (m/s)	308.9 (± 64.5)	316.5 (±135.5)		0.853
E/A ratio of tricuspid valve	1.21 (±0.34)	1.45 (±0.44)		0.240
E/E’ ratio of tricuspid valve	12.7 (±2.38)	10.05 (±0.92)		0.087

*EMH* extramedullary hematopoiesis, *LVH* left ventricular hypertrophy, *LDH* lactic acid dehydrogenase

**Table 4:** Multivariable analysis of chronic urine NGAL > 5 ng/ml

Factor associated with chronic urine NGAL > 5 ng/ml	Odds Ratio (OR)	*95% confidence interval*	*p*-value
Female	8.05	1.99–32.64	< 0.01
Splenectomy	0.55	0.16–1.90	0.35
EMH	3.64	0.90–14.66	0.07
Combined DFO + DFP	2.60	0.80–11.10	0.11
Hemochromatosis	2.01	0.67–6.05	0.22
24 h. urine protein	1.00	0.99–1.01	0.18
24 h. urine magnesium	1.38	0.99–1.93	0.06
Cardiorenal syndrome	2.57	0.37–17.90	0.34
Presence of heart failure	0.42	0.73–2.44	0.34
Elevated serum LDH	1.80	0.56–5.71	0.32

*EMH* extramedullary hematopoiesis, *DFO* deferoxamine, *DFP* deferiprone, *LDH* lactic acid dehydrogenase

A binary logistic-regression model was performed in the case of the multivariable analysis and confirmed an independent association between CRS with the type of thalassemia [β^0^/β^E^ VS. β^0^/β^0^ thalassemia (OR = 0.005, *p* = 0.002)], presence of EMH (OR 20.549, *p* = 0.016), presence of PHT (OR 25.455, *p* = 0.016), elevated serum NT-proBNP (OR 1.028, *p* = 0.022) and elevated 24-h urine magnesium (OR 1.913, *p* = 0.016) as shown in Table 5. There was no association between CRS and sex, age, splenectomy, transfusion status, mean hemoglobin level, mean serum ferritin, liver iron concentration, serum LDH, thyroid function, serum cortisol, hypogonadism, all echocardiogram parameters, cardiac T2* and type of iron chelation.

**Table 5:** Multivariable analysis showing association with Cardiorenal syndrome

Factor associated with CRS	Odds Ratio (OR)	*95% confidence interval*	*p*-value
Thalassemia type			
β^0^/β^E^ VS β^0^/β^0^ thalassemia	0.005	0.001–0.139	0.002
β^0^/β^E^ thalassemia VS Hemoglobin H disease	0.003	0.001-NA	0.714
Splenectomy	8.287	6.71–102.39	0.099
Extramedullary hematopoiesis	20.549	1.77–238.72	0.016
Hemochromatosis	3.078	0.45–21.10	0.252
Pulmonary hypertension	178.1	8.15–3893.0	0.001
Increased serum NT-proBNP	1.028	1.01–1.05	0.022
24 h urine protein	0.995	0.99–1.00	0.142
24 h urine magnesium	1.913	1.13–3.24	0.016

*CRS* Cardiorenal syndrome

## Discussion

Cardiac complications are common in cases of thalassemia. The two most common manifestations are biventricular dilated cardiomyopathy and arrhythmia [7, 26, 27, 29]. In TDT patients, the prevalence of cardiac abnormalities has been reported as follows: iron overload 44%; LV dysfunction 8–19%; increased cardiac output/index 60%; abnormal ECG 46% (T wave abnormalities 34% and right bundle-branch block 12%); history of acute myocarditis 4.5% and heart failure 2.5–4% [3, 26, 30]. Elevated serum levels of NT-proBNP (> 125 ng/mL), compared with the normal population, have also been demonstrated in TDT patients, a condition which is known to show an association with diastolic dysfunction [31–34]. In NTDT, Aessopos et al. [27] reported cardiac involvement including: congestive heart failure 5.4%; history of acute pericarditis 8.1%; pericardial thickening 34.5%; leaflet thickening 48%; endocardial calcification 20.9%; left-sided valve regurgitation (aortic 15.4%, mitral 47.2%), and pulmonary hypertension (peak systolic tricuspid gradient > 30 mmHg) 59.1%. In addition, all patients had a high cardiac output and normal LV contractility.

Renal abnormalities in thalassemia manifest as tubular dysfunction and glomerular hyperfiltration. One mechanism associated with tubular dysfunction in thalassemia is due mainly to renal tubular cell hypoxia caused by chronic anemia that contributes to tubular cell death and fibrosis. Another contributory factor is the occurrence of iron deposition in glomeruli, proximal tubules and the interstitium which results in glomerulosclerosis, tubular atrophy, and interstitial fibrosis [35]. Evidence of tubular dysfunction includes hypercalciuria (12.9–22%), proteinuria (8.6–89%), phosphaturia (9.2%), magnesiumuria (8.6%), hyperuricosuria (38–82.4%) and microalbuminuria (29%) [28, 36, 37].

Thalassemia is a highly prevalent condition in countries of Southeast Asia including Thailand, and currently there is restricted data regarding the coexistence of renal and cardiac abnormalities or CRS with the condition. This study has demonstrated that the prevalence of CRS in thalassemia is 27.8%. To the best of our knowledge, our study was the first study that has demonstrated the prevalence of CRS in thalassemia. The occurrence of CRS showed an association with the type of thalassemia (β^0^/β^0^ thalassemia), EMH, PHT, increased 24-h urine magnesium and elevated serum NT-proBNP. However, the cut points for BNP and NT-proBNP in our study were below those set for those with CKD and heart failure in previous studies [38, 39]. An association between CRS and EMH/ PHT may be explained by the presence of chronic severe anemia with inadequate transfusion which resulted in cardiac and renal hypoxia and dysfunction manifested by elevated serum NT-proBNP levels and magnesiumuria. Chronic anemia-associated systemic hypoxia generally enhances cardiac compensation by initially increasing cardiac output which ultimately leads to pathologic cardiac remodeling such as chamber enlargement if the anemia is not corrected. Chronic activation of the renin-angiotensin-aldosterone system (RAAS) may be a major key to treating secondary CRS in thalassemia patients since cardiac and renal hypoxia generally stimulates RAAS. It is noteworthy that persistent RAAS activation can induce myocardial fibrosis, renal tubular damage, efferent arteriole constriction causing glomerular hypertension, proteinuria and renal fibrosis, thereby leading to CRS [40].

The degree of tissue hypoxia may explain why CRS showed an association with β^0^/β^0^ thalassemia. β^0^/β^0^ thalassemia is caused by total deletion of the beta globin gene resulting in a severe misbalance of the alpha and beta globin chains and severe anemia. β^0^/β^E^ thalassemia sufferers however, have less severe anemia than β^0^/β^0^ thalassemia because some beta globin chains, despite being a defective form, are still produced. Although hemoglobin E is an abnormal form of hemoglobin and made of abnormal globin chains, it can still carry and deliver oxygen to tissues.

Neutrophil gelatinase-associated lipocalin (NGAL) is a 25 kDA, low molecular weight protein, majorly found in neutrophil granules and renal tubular cells. NGAL is involved in iron transportation, iron binding and renal cell repair. It passes freely through the glomeruli then is for the most part reabsorbed in the proximal renal tubules. NGAL can be elevated in inflammatory conditions and renal diseases such as: autosomal dominant polycystic kidney disease; immunoglobulin A nephropathy; HIV nephropathy; contrast-induced nephropathy; urinary tract infections and renal tubular injury. Elevation of urine NGAL is associated with decreased estimated GFR, urinary albumin excretion, increased serum NT-proBNP levels and may be potentially used for early detection of AKI [41, 42]. However, several studies have shown inconclusive results as regards urine NGAL as a predictor of CKD progression [43–48].

Clinical studies in cardiac surgery settings, after contrast infusion, critical illness, and traumatic patients, have demonstrated an association between NGAL and early detection of AKI. Both urine and serum NGAL have been shown to be elevated preceding the elevation of serum creatinine in AKI patients. Elevation of urine or serum NGAL has also been observed in CRS patients, especially in cases of type 1 CRS, and may have been beneficial in the diagnosis of other types [18, 49–53]. However, recent large prospective cohort study AKINESIS has shown serum and urine NGAL was not superior to serum creatinine for predict worsening renal failure or need for renal-replacement therapy in patients with acute heart failure (CRS type 1) [54–56].

A study of relevant literature shows the cutoff level point for the level of urine NGAL for AKI or CKD progression varies from 10 to 500 ng/ml depending on patient population and conditions but to date there is no generalized standard cutoff for urine NGAL [21, 41, 42, 49, 57]. In our study, the univariate analysis indicated that a chronic urine NGAL level higher than 5 ng/ml showed an association with female gender, combined deferoxamine (DFO) and deferiprone (DFP) treatment, hemochromatosis, elevated serum LDH, chronic proteinuria, and increased 24-h urine protein. Proteinuria in thalassemia may be contributed to by glomerular or tubular injury, but evidence from our study supports tubular injury to be the main cause as urine NGAL was a tubular damage marker and was not elevated in cases of glomerular injury [58, 59]. The cut-off of urine NGAL in this study quite low compared to other studies and we hypothesized that thalassemia patients had glomerular hyperfiltration which can lower urine NGAL causing the low cut-off. The association between hemochromatosis and urine NGAL excretion may be due to iron toxicity that produces free radicals to injure renal tubular cells. Combined use of iron chelators, DFO and DFP, results in a greater severity of iron overload and renal tubular injury. This may explain its association with chronic urine NGAL excretion. In addition, both DFO and DFP may be directly toxic to renal tubular cells as they synergistically enhance iron excretion via the kidney. This therapy also results in increased iron accumulation in renal tubular cells which causes tubular cell damage [13, 60]. In this study we found that the threshold of > 5 ng/mL was associated with CRS. However the threshold of urine NGAL in our study was quite low compared to other studies [21, 61]. As previously mentioned, the cut point of urine NGAL can vary depending on patient population. But all studies were in agreement that NGAL could be a useful marker for predicting tubular damage and AKI [60].

A limitation of this study is that it is a cross-sectional study with only a 3 month follow-up. Prospective long term follow-up would quite possibly detect more detailed dynamics of the disease and other complications, in particular those related to renal abnormalities and changes in eGFR. Moreover, a prospective long term study or a study with increased follow-up times and shorter intervals may be useful in pinpointing more precise onset of cardiac and renal abnormalities in order to indicate a specific type of CRS. In this study, data collection of renal abnormalities was based on laboratory criteria while renal symptoms may have not developed at that point. Echocardiographic data in our study also had some missing data. The majority of cases of renal abnormalities in this study were characterized by proteinuria which is common in type 2 CRS, and can be found in types 4 and 5 CRS.

Apart from conventional renal markers such as serum creatinine and eGFR, the tests for which are not sensitive enough for early detection of renal injury, only a single novel kidney injury biomarker, urine NGAL, was used in the present study. Using a panel of biomarkers may give a higher level of sensitivity for the detection of renal abnormalities. One interesting alternative marker which provides an early indication of renal damage is ‘renal functional reserve’ as some thalassemia patients tend to have glomerular hyperfiltration which may reflect a functional reserve response to stress. Chronic stress to the kidney could result in low renal functional reserve despite a normal eGFR or serum creatinine level [62]. However, the cut-off of urine NGAL in this study was quite low compared to levels described in previous studies. These may have caused false positive readings of elevated urine NGAL. Urine NGAL as a standalone measure is not a precise indicator for a diagnosis of kidney injury. The inclusion of other markers such as TIMP-2, KIM-1 in addition to NGAL in the future could improve the precision. Renal ultrasound was not routinely done in all patients unless there was a clinical indication/suspicion which may have resulted in structural diseases of the kidneys and urinary tract as well as kidney stones being missed.

There were also two important confounding factors in this study into CRS in thalassemic patients. First, iron chelation therapy especially deferasirox can cause renal failure and proteinuria. However, this factor would be unlikely to impact on the findings of our study since only one patient had an eGFR below 60 ml/min/1.73 m^2^. Second, heart failure symptoms can mimic anemic symptoms, but to prevent this potential misinterpretation we used physical examination to ensure the diagnoses were accurate. We also recorded symptoms after blood transfusion, which should be improved if the patient had symptoms indicating anemia.

## Conclusions

CRS is relatively common in thalassemia patients, especially in cases of β^0^/β^0^ thalassemia. This condition is associated with the presence of EMH and PHT. Elevated serum NT-proBNP and magnesiumuria may be useful markers for the detection of CRS in thalassemia cases.

## Supplementary information

**Additional file 1: Table S1.** Criteria for diagnosis of heart failure (all 3 criteria required). **Table S2.** Cardiac and renal abnormalities (*n* = 90).

## Data Availability

The data that support the findings of this study are available from the corresponding author, AT, adisak.tan@cmu.ac.thupon reasonable request. The data are not publicly available due to privacy or ethical restrictions.

## Abbreviations

CRS: Cardiorenal syndrome
EMH: Extramedullary hematopoiesis
OR: Odds ratio
TDT: Transfusion dependent thalassemia
NTDT: Non-transfusion dependent thalassemia
MRI: Magnetic resonance imaging
LIC: Liver iron concentration
CBC: Complete blood count
BUN: Blood urea nitrogen
SCr: Serum creatinine
LDH: Lactate dehydrogenase
TFT: Thyroid function test
eGFR: estimated glomerular filtration rate
NGAL: Neutrophil gelatinase-associated lipocalin
HFpEF: Heart failure with a preserved ejection fraction
HFmrEF: Heart failure with a mid-range ejection fraction
HFrEF: Heart failure with a reduced ejection fraction
KDIGO: Kidney Disease Improving Global Outcomes
AKI: Acute Kidney Injury
AER: Albumin excretion rate
ACR: Albumin to creatinine ratio
Cr: Creatinine
BW: Body weight
PHT: Pulmonary hypertension
SLE: Systemic lupus erythematosus
RAAS: Renin-angiotensin-aldosterone system
