# Supplementary material for: Cardiorenal syndrome in thalassemia patients
Source: BMC Nephrol. 2020 Aug 3;21:325. doi: 10.1186/s12882-020-01990-8 (PMC7398251; doi:10.1186/s12882-020-01990-8)
Supplement: Supplementary file 1 — Additional file 1: Table S1. Criteria for diagnosis of heart failure (all 3 criteria required). Table S2. Cardiac and renal abnormalities (n = 90). [file 12882_2020_1990_MOESM1_ESM.docx]

**Supplementary appendix**

**Table S1** Criteria for diagnosis of heart failure (all 3 criteria required)

| Criteria | HFrEF | HFmrEF | HFpEF |
| --- | --- | --- | --- |
| 1 | Symptoms +/- signs* | Symptoms +/- signs* | Symptoms +/- signs* |
| 2 | LVEF < 40% | LVEF 40-49% | LVEF 50% |
| 3 | - | 1. Elevated natriuretic peptides**  2. One of the following  2.1 relevant structural heart disease (LVH and/or LAE)  2.2 diastolic dysfunction | |

* Signs may be not found in early heart failure, especially HFpEF, and in patients with previous diuretic use.

**Cutoff, BNP > 35 pg/ml, NT-proBNP > 125 pg/ml

**Table S2** Cardiac and renal abnormalities (n = 90)

|  | CRS =25  n (%) | No CRS =65  n (%) |
| --- | --- | --- |
| Cardiac abnormalities  Structural cardiac abnormalities  LVH  RVH  LAE  RAE  Heart failure  Diastolic dysfunction | 25 (27.8)  25 (27.8)  15 (16.7)  13 (14.4)  13 (14.4)  7 (7.8)  8 (8.9)  4 (4.4) | 10 (11.1)  10 (11.1)  6 (6.7)  3 (3.3)  4 (4.4)  2 (2.2)  0 (0)  0 (0) |
| Renal abnormalities  Chronic proteinuria  eGFR < 60 | 25 (27.8)  24 (26.7)  1 (1.1) | 27 (30)  27 (30)  1 (1.1) |

CRS = Cardiorenal syndrome; LVH = left ventricular hypertrophy; RVH = right ventricular hypertrophy; LAE = left atrial enlargement; RAE = right atrial enlargement; eGFR = estimated glomerular filtration rate

**Table legend**

Table S1 Criteria for diagnosis of heart failure (all 3 criteria required)

Table S2 Cardiac and renal abnormalities
